# Proteomic Response and Quality Maintenance in Postharvest Fruit of Strawberry (*Fragaria* × *ananassa*) to Exogenous Cytokinin

**DOI:** 10.1038/srep27094

**Published:** 2016-06-01

**Authors:** Li Li, Dongdong Li, Zisheng Luo, Xinhong Huang, Xihong Li

**Affiliations:** 1 College of Biosystems Engineering and Food Science, Zhejiang Key Laboratory for Agro-Food Processing, Zhejiang University, Hangzhou 310058, People’s Republic of China; 2 Key Laboratory of Food Nutrition and Safety (Ministry of Education), Tianjin University of Science and Technology, Tianjin 300457, People’s Republic of China

## Abstract

The limitations in current understanding of the molecular mechanisms underlying fruit response to the application of plant growth regulators have increasingly become major challenges in improvement of crop quality. This study aimed to evaluate the response of strawberry to the preharvest application of exogenous cytokinin known as forchlorfenuron (CPPU). Postharvest internal and physiological quality attributes were characterized following storage under different conditions. Hierarchical clustering analysis via a label-free proteomic quantitative approach identified a total of 124 proteins in strawberries across all treatments. The expression profiles of both proteins and genes spanned the ranged role of cytokinin involved in primary and secondary metabolism, stress response, and so on. Eighty-eight proteins and fifty-six proteins were significantly regulated immediately at harvest and after storage, respectively. In general, the glycolysis in strawberry was only regulated by CPPU before storage; in addition to the accelerated photosynthesis and acid metabolism, CPPU application maintained higher capacity of resistance in strawberry to stress stimuli after storage, in comparison to control. Nevertheless, the volatile biosynthesis in strawberry has been suppressed by exogenous CPPU. Novel cytokinin response proteins and processes were identified in addition to the main transcriptomic expression to gain insights into the phytohormone control of fruit postharvest quality.

The discovery of plant growth regulators (auxins, cytokinins and gibberellins) has provided new insights into promoting cell division and expansion, enlarging fruit, retarding leaf senescence, and increasing crop yield^1^. Forchlorfenuron (CPPU; FCF; *N*-(2-chloro-4-pyridyl)-*N*-9-phenylurea) is a cytokinin-like plant regulator with strong activity and can be absorbed by fruits, flowers, stems and roots^2^. Preharvest application of CPPU delayed the maturation and ripening of grape, pineapple, cherry, and kiwi^3^,^4^. CPPU can also increase the fruit set, yield, and size after preharvest treatment and reduce deterioration to maintain fruit quality during postharvest storage^5^. Prebloom application of CPPU increases the fruit set, whereas postbloom application affects the fruit size, color, maturity, and storage quality^6^. Although the effectiveness of plant growth regulator application has been relatively, widely, and easily evaluated in terms of fruit enlargement, the same effects do not apply for biochemical attributes.

It has been reported that the plant growth regulator could enhance the chlorophyll concentration and CO_2_ assimilation rate, thereby increasing the assimilated supply to the fruits^7^,^8^. CPPU application was reported to exert good effects on banana and broccoli, such as inhibited respiration rate and natural browning, as well as delayed maturity and softening^2^. However, the molecular mechanisms underlying the role of exogenous cytokinin in postharvest quality performance of strawberry remain poorly understood.

Recently, substantial research effort has been devoted to study the relationship among ripening^9^,^10^, volatile composition (Defilippi *et al.* 2009; González *et al.* 2009; Raab 2006), antioxidant capacity^11^, postharvest treatment^12^, and fruit quality of strawberry at the physiological level. Despite the recent sequencing of 10,825 ESTs and 33,264 coding genes from *Fragaria vesca*^13^, genomic information on strawberry is rarely reported. The existing genomic data are insufficient to completely characterize the molecular machinery and reflect the dynamic state in fruit cell because the expression profiles of the final gene products cannot be accurately predicted at the transcriptomic or genomic level alone^14^. Therefore, proteomics has emerged as a new platform that allows faster discovery and more sensitive and accurate profiles; this platform has also become a powerful tool to identify and qualify proteins present in biological systems^15^. To date, few proteomic studies have been conducted to correlate proteome profiles with strawberry ripening^16^,^17^, strawberry allergens^18^ and varietal differences^19^.

In the current study, the proteomic approach was used to elucidate the role of cytokinin in the variation of strawberry postharvest performance at the molecular level. The interactions between postharvest quality attributes recommended by Mitcham *et al.*^20^) and the proteomic profiles, as well as the expression profiles of major genes involved, were discussed within the context of fruit response to exogenous application of CPPU.

## Results and Discussion

### Residue and dissipation of CPPU in strawberry

The residue and dissipation behavioral characteristics of CPPU applied at three different concentrations were presented in Table 1. In all cases, the dissipation rate was fast in the beginning after CPPU application but slowed down with the passage of time. The dissipation process of CPPU in strawberry after treatment followed the first-order kinetic reaction instead of the non-linear pattern. From these results, the CPPU in strawberry degraded faster with the higher initial deposit of CPPU treatment. Approximately 54%, 49%, and 34% of the initial deposits of CPPU at concentrations of 5, 10, and 15 mg L^−1^, respectively, were dissipated within the seventh day. The results indicated that the residue of CPPU collected in strawberry samples was below the Maximum Residue Levels in Europe (EU-MRL) of 0.01 mg kg^−1^ within 25 d after preharvest CPPU application.

The half-life of CPPU in strawberry was 4.4–7.2 d (Table 1). Previous research demonstrated that the half-life of CPPU was 15.8–23.0 d in citrus^21^, 11.6–23.1 d in grape^22^, and 1.2–1.7 d in watermelon^23^. The differences in half-life among crops can be attributed to the physicochemical properties of plants, such as water content, acid/base characteristics, weather conditions as well as the potential degradation or biosynthesis by enzymes in fruits, such as cytokinin oxidase or cytokinin dehydrogenase.

### Physiological quality traits

**Figure 1:**
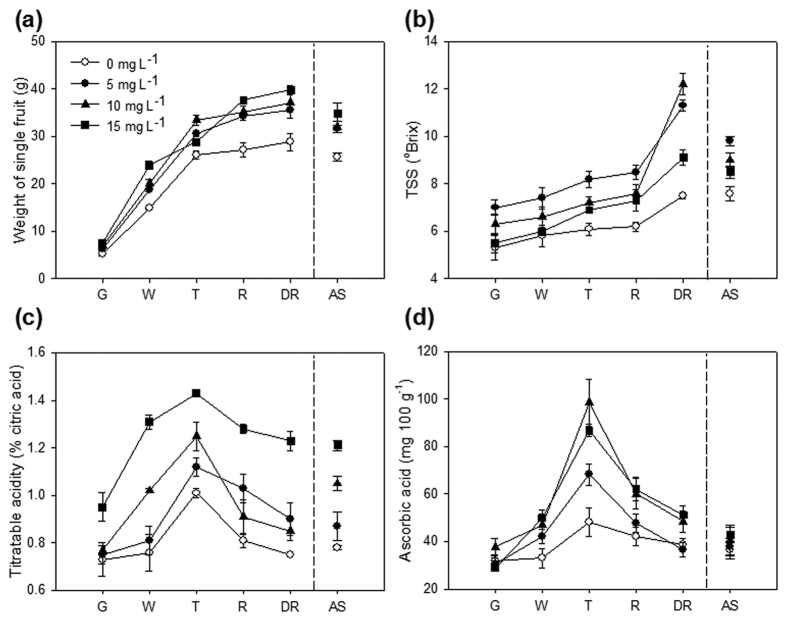
Postharvest physiological quality traits of strawberry in response to CPPU application. (**a**) Weight of single fruit; (**b**) total soluble solids (TSS); (**c**) titratable acidity; (**d**) ascorbic acid. G, green fruit; W, white fruit; T, turning fruit; R, firm and ripe fruit; DR, dark red fruit with senescence. Data shown are mean ± standard deviation (*n* = 3).

**Figure 2:**
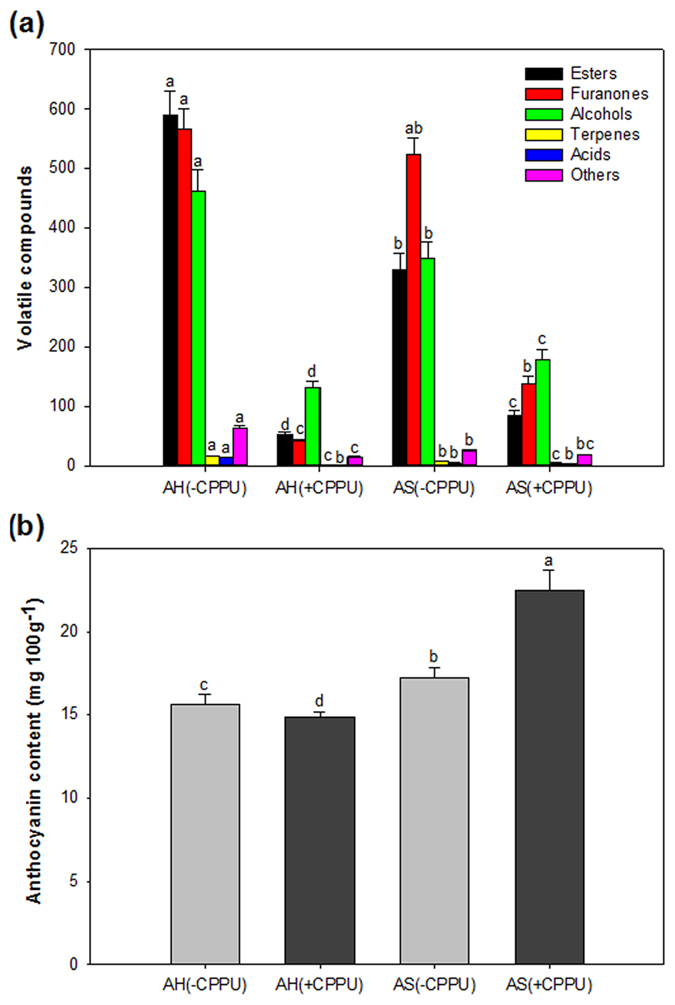
Distribution of volatile categories (**a**) and anthocyanin concentration (**b**) in strawberry in response to CPPU application in strawberry at harvest (AH) and after storage (AS). G, green fruit; W, white fruit; T, turning fruit; R, firm and ripe fruit; DR, dark red fruit with senescence. Data shown are mean ± standard deviation (*n* = 3). Different lowercase letters represent statistical significance (*P* < 0.05).

To link the proteomic profiles with changes in physiological quality of strawberry in response to exogenous CPPU application, quality traits including the fruit weight, total soluble solid (TSS), total acidity and ascorbic acid content of strawberry during ripening and senescence, as well as the production of volatiles and anthocyanins, were investigated in this study (Figs 1a–d and 2a,b).

The development of the weight of individual strawberry fruit at different maturity stages, green fruit (G, medium-size green strawberry, approximately 14 d after anthesis), white fruit (W, no sign of pigment, approximately 19 d after anthesis), turning (T, half of each strawberry is colored red, approximately 24 d after anthesis), red (R, firm and ripe strawberry, approximately 29 d after anthesis), and dark red (DR, strawberry with senescence, after 34 d after anthesis) followed the first-order kinetic reaction (Fig. 1a). CPPU treatments remarkably increased the fresh weight (FW), particularly the application of increased dose at 10 or 15 mg L^−1^. The CPPU application enhanced the fruit weight of strawberry by 21.0–42.9% compared with that of the untreated fruit.

The TSS content increased during strawberry ripening as expected because of the relatively high metabolism observed in ripened strawberry; the strawberry samples responded to CPPU application, and treatment with 5 mg L^−1^ exhibited the highest TSS content during ripening (Fig. 1b). TSS increased from the initial 5.3 and 7.0 °Brix to the final values of 7.5 and 11.3 °Brix in the control and 5 mg L^−1^ CPPU-treated samples during ripening, respectively. Application of exogenous cytokinin at lower concentration (5 mg L^−1^) could impel the accumulation of sugars in early developmental stage, and the cytokinin regulated the metabolism of endogenous hormone in fruits, which also contributed to the increase of sugar concentration in late developmental stage. However, the fruit enlargement, which was induced by the CPPU application at higher concentrations (10 and 15 mg L^−1^), delayed the capacity of carbohydrate transportation, resulting in the decrease of sugar content in fruit. In addition, the exogenous CPPU application at excess concentration (such as 15 mg L^−1^) and/or for excess time was dedicated to postpone the fruit maturation and senescence, which was another reason of the sugar decrease. CPPU application at 10 mg L^−1^ remarkably increased the TSS content from 6.3 °Brix to 12.2 °Brix, with the highest TSS content in dark red strawberry. A similar increasing trend of TSS was observed in CPPU application on *Actinidia deliciosa*^24^. By contrast, the TSS in hardy kiwifruit slightly but significantly decreased, whereas the ratio of TSS/titratable acidity was significantly increased by CPPU^4^. In addition, the TSS in postharvest grape clusters^25^ and banana^2^ was significantly reduced by CPPU application. Nevertheless, the TSS in ‘Hayward’ kiwifruit was not significantly affected by pre-anthesis CPPU application at harvest and after storage^26^.

The titratable acidity in strawberry gradually increased during early ripening and reached the highest value at the turning stage (T) then decreased over the late ripening process (Fig. 1c). The doses of CPPU can significantly decrease the level of titratable acidity in strawberry during ripening. Among all the treatments, the plants treated with 10 mg L^−1^ CPPU exhibited higher titratable acidity, whereas those treated with 5 mg L^−1^ CPPU exhibited lower titratable acidity. The regulation of titratable acidity in strawberry by CPPU application in the present study was consistent with the results in hardy kiwifruit^4^. Furthermore, the titratable acidity in postharvest grapes was previously reported to be induced by CPPU application^25^.

The ascorbic acid content in strawberries increased from green to turning stage and then decreased from turning to dark red stage (Fig. 1d). No significant differences were demonstrated in strawberry samples harvested at the G, W, and DR stages, whereas the CPPU application significantly enhanced the biosynthesis of ascorbic acid in strawberries harvested at the turning and red stages. Given the CPPU application at 10 mg L^−1^ as an example, the ascorbic acid content was increased to almost two times as that of control at turning stage. It has been reported that the accumulation of isoflavones evidently showed a positive correlation with the application of cytokinins^27^. By contrast, the total ascorbic acid concentration in hardy kiwifruit was significantly decreased by CPPU application^4^, and the ascorbic acid content in ‘Hayward’ kiwifruit was not significantly affected by pre-anthesis CPPU application^26^.

The aroma volatiles of strawberry consist of a large number of substances belonging to different chemical classes including esters, alcohols, acids, terpenes, and furanones. The main volatile compounds of the ‘Akihime’ strawberries examined in the present study were esters, alcohols, and furanones (Fig. 2a and Supplementary Table S1). Overall, CPPU application significantly decreased the total volatile content of strawberry by 85.7% and 65.3% compared with the controls before and after storage, respectively. The significant reduction of esters and alcohols was accompanied by the increase of TSS in red strawberries responded to CPPU application both before and after storage (Figs 1b and 2a). The production of esters and alcohols in strawberry were synergistically derived from the glycolysis pathway as well as the fatty acid metabolism^28^. Our results demonstrated that the exogenous CPPU might inhibit the flux from the total sugars to specific esters and alcohols in ‘Akihime’ strawberry.

In addition, the anthocyanin concentration in strawberries increased by 10.5% and 51.4%, respectively, in control and CPPU-treated strawberries after storage (Fig. 2b). The results revealed that the exogenous cytokinin was involved in the regulation of anthocyanin metabolism and CPPU significantly induced the anthocyanin biosynthesis in present study.

### Hierarchical clustering of protein profiles and gene expression in response to CPPU

**Figure 3:**
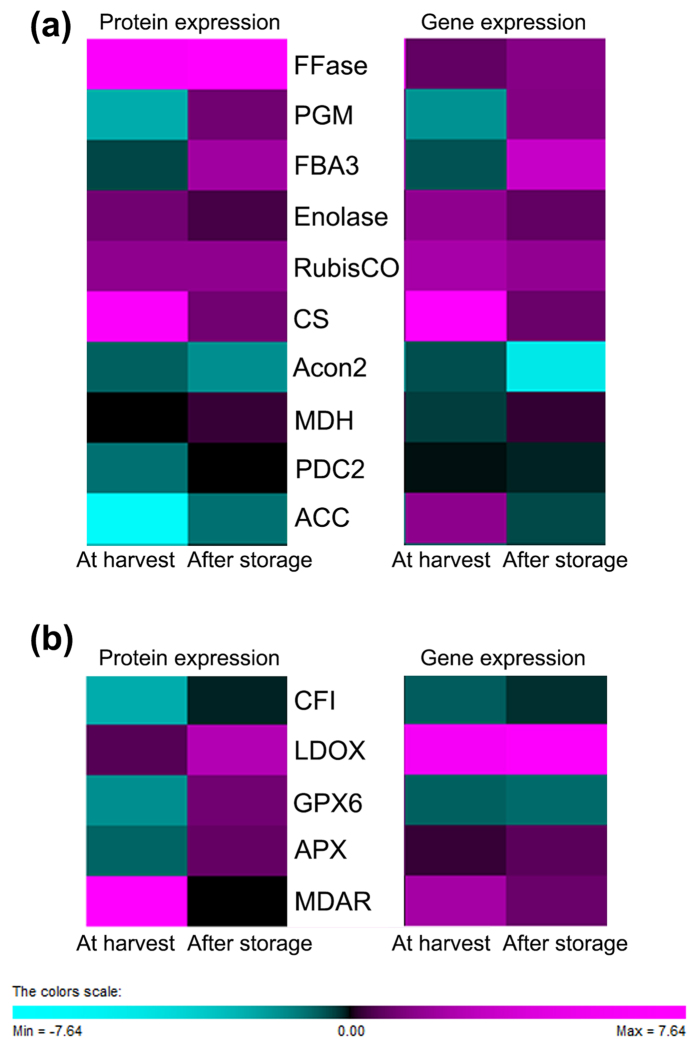
Relative expression of proteins and genes involved in the primary and volatile metabolism (**a**) as well as stress response (**b**) in strawberry at harvest (AH) and after storage (AS) proteins in response to CPPU application. The detailed relative expression level of related genes is shown in Supplementary Figure S1–S3.

**Figure 4:**
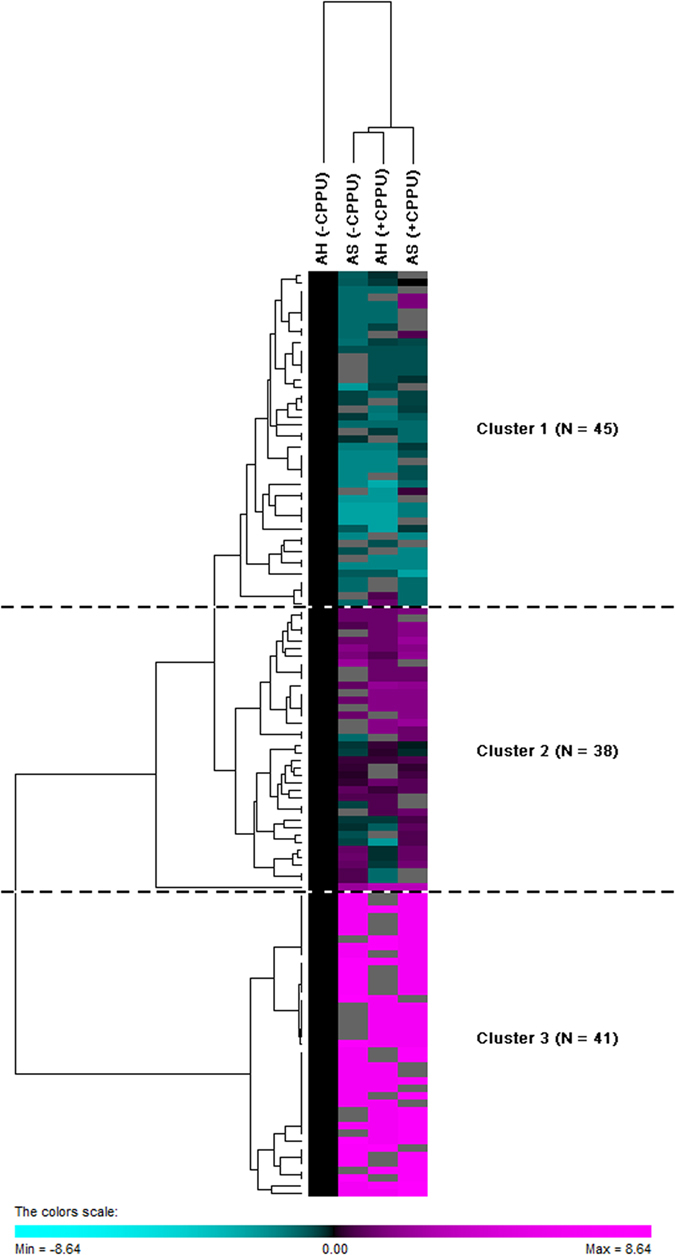
Hierarchical clustering analysis of 124 strawberry fruit proteins that responded to CPPU application at harvest (AH) and after storage (AS). The detailed identification and qualification information of the proteins are listed in Supplementary Table S2. The increased intensity of magenta or blue colors indicate the differentially increased or decreased abundance compared with controls [AH(–CPPU)]. Three major protein clusters were formed based on the abundance patterns across different treatments.

In the past few years, the identification and quantification of proteins have made significant progress. Current focus is shifting to the elucidation of biological processes in plants at the proteomic level. To investigate the proteomes regulated by preharvest CPPU treatment, proteins extracted from strawberries without CPPU application at harvest were used as denominators against the ratios of normalized spectral counts of proteomes from the CPPU-treated strawberries after storage and were compared in the present study. Based on the patterns of relative abundance, relative expression of genes involved in the primary and volatile metabolism as well as stress response in strawberry at harvest and after storage were studied (Fig. 3 ). With regard to the proteome, hierarchical clustering analysis identified three clusters among the 124 common proteins present in strawberry across all treatments before and after storage (Fig. 4). By using a two-fold change as the variance for an upshift or downshift in abundance, the normalized ratio of protein abundance changes revealed significant and dynamic changes in protein profiles. On the basis of the relatively regulated expression patterns, the hierarchical clustering analysis identified three clusters among the 124 common proteins present in strawberry across all treatments (Fig. 4). Overall, 45, 38, and 41 proteins were included in Clusters 1, 2, and 3, respectively. The relative expression levels of all proteins are listed in Supplementary Table S2.

**Figure 5:**
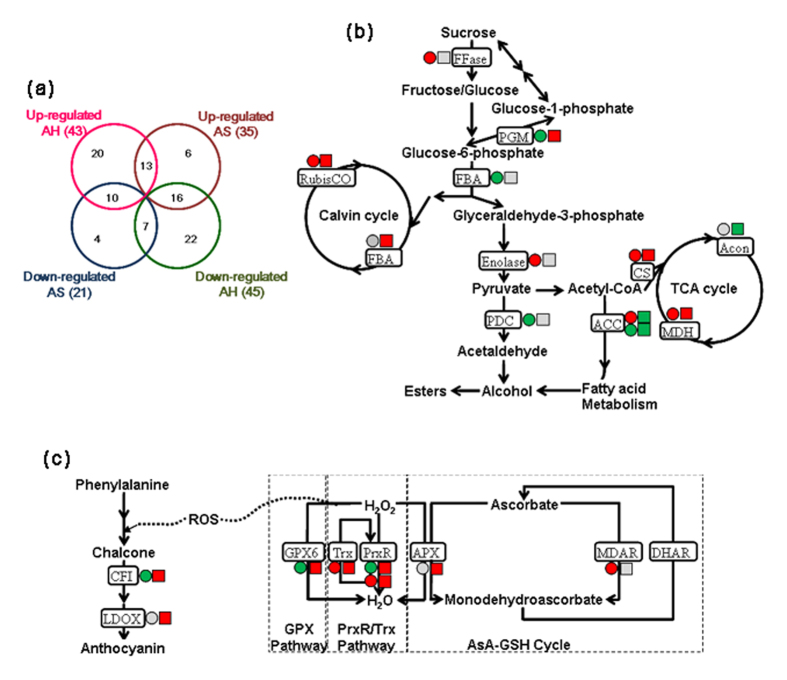
(**a**) Venn diagram of the number of identified proteins in the strawberry at harvest (AH) and after storage (AS) in response to CPPU application. Schematic diagram of the primary metabolism (**b**) and the stress response system (**c**) of strawberry fruit after preharvest CPPU application. The expression of each protein in the red/magenta and green/blue lines indicated the expression levels of proteins significantly up-regulated and down-regulated by CPPU application, respectively, at harvest (⦁) and after storage (◾). The grey color indicated that the expression level was not significantly regulated, and the color brightness is proportional to the expression ratio.

The up-regulated and down-regulated proteomes at harvest and after storage are presented in a Venn diagram (Fig. 5a). Given the changes in protein expression among treatments, 43 and 45 proteins were up-regulated and down-regulated in strawberry at harvest, respectively. By contrast, 35 and 21 proteins were up-regulated and down-regulated by CPPU after storage, correspondingly. Up to 13 proteins were significantly up-regulated, whereas 7 proteins were significantly down-regulated in response to CPPU both before and after storage. The differences in abundance of these proteins illustrated the complex regulatory network involved in the active processes of postharvest strawberry in response to preharvest CPPU application.

### Proteins associated with primary metabolism and volatile biosynthesis

The results indicated that the proteins involved in the biosynthesis of primary metabolites were differentially regulated by CPPU application at harvest and after storage (Fig. 5b). Proteomic evidence has been provided for the complex nature of sugar and acid metabolic networks, which are major contributors to fruit quality during ripening and senescence^28^. At fruit harvest, β-fructofuranosidase (FFase; No. 30 in Cluster 3) and enolase (No. 24 in Cluster 2) were up-regulated, whereas phosphoglucomutase (PGM; No. 33 in Cluster 1) and fructose-bisphosphate aldolase (FBA; No. 1 in Cluster 3) were down-regulated in response to preharvest CPPU application. The abundance of three proteins involved in the glycolytic pathway, namely, FFase, FBA, and enolase, did not significantly vary after storage. PGM can catalyze the reversible conversion of glucose-1-phosphate into glucose-6-phosphate. As the only exception, PGM showed a noteworthy reduction at harvest, whereas a mild increase was regulated by CPPU after storage. The expression of all these genes was evidently consistent with protein expression, with the exception of FFase, which was only four times higher at the proteomic level than the gene expression level (Fig. 3a). The glycolysis in strawberry before storage was affected by CPPU application, but most proteins involved in glycolysis were not regulated after storage. The synergistic regulation of these proteins was partially contributed to the increase in TSS contents detected, which showed the increases of 11.8–37.1% and 11.2–28.9% in CPPU-treated red strawberries at harvest and after storage, respectively, compared with untreated samples (Fig. 1b).

Furthermore, two proteins related to the carbon fixation in strawberry were up-regulated by CPPU, including two enzymes involved in the Calvin cycle: the RuBisCO protein (No. 14 in Cluster 2) and chloroplastic like FBA 3 (No. 16 in Cluster 1). The induction of RuBisCO at the protein and gene levels (Fig. 3a) suggested that the stress of senescence impede photosynthesis by accelerating the Calvin cycle, as well as causing protein denaturation and DNA mutations^29^. These findings were consistent with previous results that the RuBisCO protein was up-regulated by plant growth regulators called gibberellins in Japanese apricot flower buds^30^ and rice leaf sheath^31^. The RuBisCO protein in rice lamina joint was increased by the plant growth regulator brassinosteroid^31^. In addition, the up-regulated level of FBA protein by CPPU in strawberry agreed with the increased expression of FBA by gibberellins in rice roots^32^.

Citric acid is the predominant organic acid in strawberry^17^. The malate dehydrogenases (MDHs, No. 23 and No. 32 in Cluster 2) and aconitate hydratase 2 (Acon, No. 41 in Cluster 1) involved in the tricarboxylic acid (TCA) cycle have also been identified in strawberry. Likewise, the citrate synthase (CS; No. 32 in Cluster 3) of strawberry, which connects the glycolytic pathway and TCA cycle have been identified. In the TCA cycle, CS stands as a pace-making protein in the first step^33^, and MDH converts malate to oxaloacetate (the intermediate in TCA cycle) in the final step. Furthermore, Acon catalyzes the stereo-specific isomerization of citrate to isocitrate via *cis*-aconitate in the TCA cycle^34^. In the current study, the up-regulated expression of CS and MDH and the down-regulated expression of Acon (Fig. 5b) were synergistically correlated with the increased profiles of titratable acidity in response to CPPU application before and after storage (Fig. 1c). MDH has also been related to a signaling pathway that is active in response to oxidative stress^28^,^35^.

The strawberry flavor results from more than 300 volatile aromatic compounds; majority of these are esters, alcohols, aldehydes, acids, and so on^28^. With regard to the proteins related to the volatile biosynthesis identified in the present study, the pyruvate decarboxylase (PDC; No. 17 in Cluster 1) 2 protein, which catalyzes the pyruvate to acetaldehyde in fruit, was down-regulated at harvest by CPPU but without significant variation after storage (Fig. 5b). However, the PDC protein in rice lamina joint was significantly up-regulated by brassinosteroids^31^. PDC2 showed 7.16- and 7.08-fold higher expression at the proteomic and transcriptomic levels, respectively, in CPPU-treated fruit compared with controls (Fig. 3a).

In addition, acetyl-CoA carboxylase (ACC) catalyzes acetyl-CoA to produce malonyl-CoA through two ACC activities, biotin carboxylase (No. 2 in Cluster 2) and carboxyltransferase (No. 37 in Cluster 3), which showed lower expression levels in response to CPPU application. The most important function of ACC is to provide the malonyl-CoA substrate for fatty acid metabolism, which was contributed to the precursors for aroma volatile biosynthesis^36^. The total volatile production was significantly decreased to 14.3% and 34.7% by CPPU compared with the control at fruit harvest and after storage, respectively (Fig. 2a). In addition to the expression profiles of PDC and ACC genes in the present study, the down-regulation of these proteins provided the evidence for the suppression of volatile biosynthesis in response to preharvest CPPU application (Fig. 3a).

### Proteins related to phenylpropanoid pathway and stress response

The proteins involved in the phenylpropanoid pathway participate in plant response to biotic and abiotic stress stimuli have been identified (Fig. 5c). At the proteomic level, two proteins, chalcone-flavonone isomerase (CFI; No. 32 in Cluster 1) and leucoanthocyanidin dioxygenase (LDOX; No. 28 in Cluster 2), were down-regulated or unchanged at fruit harvest. Nevertheless, both proteins were up-regulated after storage (Fig. 5c). Furthermore, the up-regulated expression of LDOX gene contributed to the induction of anthocyanin content in strawberry in response to exogenous cytokinin (Fig. 2b). The results manifested that the acceleration of the phenylpropanoid pathway in strawberry was flowing into anthocyanin and flavonoid biosynthesis after storage^37^. The proteins chalcone isomerase, dihydroflavonol-4-reductase, and anthocyanidin reductase in this pathway are up-regulated after abscisic acid (ABA) treatment on grape berries before véraison^38^. By contrast, the activities of enzymes including phenylalanine ammonia lyase (PAL) and chalcone synthase in strawberry were significantly decreased by exogenous auxin or gibberellic acid application^39^. All the above mentioned results indicated that the phenylpropanoid pathway in non-climacteric postharvest fruits (such as grape and strawberry) might be affected by the plant growth regulators^40^.

The redox homeostasis has been studied as a metabolic interface between the oxidative stress perception and physiological response in plants to environmental stimuli^41^. The process was regulated by the interaction between the cellular antioxidant system and reactive oxygen species (ROS), resulted from endogenous oxidative processes^42^. The expression profile of related proteins and genes involved in stress response is shown in Fig. 3b. The ascorbate-glutathione (AsA-GSH) cycle was one of the most important cellular antioxidant systems in plants^43^. The ascorbate peroxidase (APX; No. 30 in Cluster 2) participates in the detoxification of H_2_O_2_, and its over-expression in tomato indicated the tolerance to chilling and salt stresses^44^. APX which catalyzes ascorbate to monodehydroascorbate in detoxification reactions was significantly up-regulated after CPPU treatment. However, monodehydroascorbate reductase (MDAR; No. 3 in Cluster 3) was up-regulated by approximately 200 times compared with the control at harvest (Fig. 5c). Similarly, the APX protein in rice leaf sheath was also increased by exogenous gibberellins application^31^. Therefore, the final concentration of ascorbate in fruit is determined by the balance of its biosynthesis, metabolism and recycling^28^. In the present study, the concentration of ascorbic acid in strawberry was partially explained by the regulation of proteins involved (Fig. 1d).

Notably, one thioredoxin H-type protein (No. 12 in Cluster 2), one 1-Cys peroxiredoxin (No. 40 in Cluster 3), and one peroxiredoxin-2B-like protein (No. 25 in Cluster 1) were up-regulated by CPPU at harvest and after storage. Another peroxiredoxin-2B-like protein was downregulated before storage while up-regulated by CPPU after storage (Fig. 5c). Peroxiredoxin (PrxR) decomposes the H_2_O_2_ that escapes from detoxification processes carried out by other enzymes such as catalase (CAT) or APX, and plays an important role in plant growth, plant development, redox signaling and ROS detoxification, as well as the protection against biotic and abiotic stress. The higher abundance of thioredoxin (Trx) and PrxR proteins in CPPU-treated samples in this study represented an important role for these proteins in redox sensing and homeostasis, to protect strawberry tissue from oxidative stress during ripening and senescence.

Another pathway that contributed to the redox system in fruits was phospholipid hydroperoxide glutathione peroxidase 6 (GPX6; No. 27 in Cluster 1), which functions to protect cells and enzymes from oxidative damage by catalyzing the reduction of hydrogen peroxide, lipid peroxides, and organic hydroperoxide^45^. GPX6 in strawberry was down-regulated to 66.7% at harvest but up-regulated by twice higher expression compared with the controls after storage.

All the above results indicated that the preharvest CPPU application can maintain higher capacity of resistance in strawberry to stress stimuli after storage, compared with the controls. In a physiological manner, the antioxidant capability and antioxidant compounds concentration in ‘Camarosa’ strawberry fruit have been reported to be increased by the preharvest auxin and gibberellic acid application^46^. Previous results reported that the gibberellins can lead to the development of oxidative stress in Japanese apricot flower buds^30^. Alternatively, the defense responses in postharvest citrus fruits were promoted by auxin-like 2,4-dichlorophenoxyacetic acid (2,4-D) application^47^; the expression of stress-related proteins in the ripening grape berries was induced by ABA treatment, with up-regulated expression of APXs^38^. The results observed in the present study confirmed the hypothesis that the application of specific cytokinin-like or auxin-like plant growth regulators can induce the defense response of fruits to biotic and abiotic stress.

## Methods

### Plant treatments

Strawberries (*Fragaria* × *ananassa* Duch. cv. ‘Akihime’) were grown in Xintai Orchard in Tianjin (latitude: 39°13′N, longitude: 117°18′E). Plants of similar size and development stage were chosen for the experiment. The fruit maturities were determined by tagging the flowers at anthesis and the CPPU (Fulmet, EC 0.1%; Kyowa, Tokyo, Japan) was sprayed on strawberry plants at concentrations of 5, 10, and 15 mg L^−1^ on the seventh day after anthesis. The distance between the double rows was 1.2 m, whereas the distance between nearby plants in double row was 0.4 m. Each row comprised one replication of each treatment, where a total of 25 plants per treatment were used for the present experiment. The control fruits were not subjected to any treatments.

After CPPU treatment, the strawberries were harvested at five selected stages of development: green fruit (G, medium-size green strawberry, approximately 14 d after anthesis), white fruit (W, no sign of pigment, approximately 19 d after anthesis), turning (T, half of each strawberry is colored red, approximately 24 d after anthesis), red (R, firm and ripe strawberry, approximately 29 d after anthesis), and dark red (DR, strawberry with senescence, after 34 d after anthesis). The fruit samples were transferred to the laboratory, sorted to discard damaged and diseased samples, then calyxes and pedicels were removed. Fruit weight was determined from twenty individual strawberries with three biological replications. The surface of the strawberries was cleaned with a 2% sodium dodecyl sulphate (SDS) solution and the red strawberries were stored at 20 ± 1 °C and 95% relative humility for 6 d of improved shelf-life. Fruit samples for analysis were immediately frozen in liquid nitrogen, placed in sealable bags and stored at −80 °C. Three independent biological replicates were prepared over harvest time using pooled tissue from twenty individual strawberries for the subsequently assay.

### Residue determination of cytokinin

The CPPU residue in strawberry was determined using the method described by Sharma^48^ with slight modifications. The CPPU residue in intact and unwashed strawberry was quantified by the HPLC system, which consisted of a Shimadzu HPLC model LC6A equipped with a variable wavelength UV–visible detector and a polymeric LiChrospher 100 RP-8 column (25 cm × 4 mm *i.d.*). The mobile phase was acetonitrile-water (55:45, v/v). The flow rate was 1.0 mL min^−1^, and detection was performed at 265 nm. Red ripe strawberries were collected at 0 (2 h after CPPU application), 5, 10, 15, 20, 25, and 32 (red ripe fruit at harvest) d after treatment and analyzed for residues of CPPU. The results were expressed as mg kg^−1^ FW, and three independent replicates were performed.

### Total soluble solids (TSS) and titratable acidity (TA)

The TSS content in the extracted strawberry juice was measured by a refractometer (ATAGO Co., Tokyo); the results were expressed as °Brix. TA was measured for each 2 mL strawberry juice sample; 0.1 mol L^−1^ NaOH was used to titrate the juice with a semi-automatic titrator (Multi-Dosimat E-415 titrator, Metrohm AG, Switzerland) to a phenolphthalein endpoint of pH 8.1. TA was expressed as the percentage of citric acid equivalents and three independent replicates were performed.

### Ascorbic acid

The concentration of ascorbic acid in strawberry was determined using the High-Performance Liquid Chromatography (HPLC)-based procedure^28^. The HPLC system used was consisted with a Hewlett Packard Series 1050 auto-sampler, a Series 1050 pump, and a Series 1040 M diode array detector. The Waters μBondapak Cl8 reversed-phase column (30 cm × 3.9 mm *i.d.*) with a Bio-Rad Bio-sil Micro-Guard ODS-5S (4.6 mm × 3 cm *i.d.*) guard column was used for separation. The results were expressed as mg 100 g^−1^ FW and three independent replicates were performed.

### Anthocyanin content measurement

Total anthocyanin contents in strawberry were determined using a previously reported method^49^. The strawberry samples were extracted with HCl/methanol and the absorbance of the supernatant after centrifuged was measured at 530 nm using a UV-visible spectrophotometer (UV-1600, Shimadzu, Kyoto, Japan). Three independent replicates were performed.

### Volatile compounds

The volatile compounds of strawberry were analyzed using the protocol described by Li *et al.*^17^ with three independent replications. Volatiles were extracted using a solid-phase microextraction (SPME) fiber (DVB/Car/PDMS, Supleco Inc., Bellefonte, USA) coated with 100 μM polydimethylsiloxane (Supelco Inc., Bellefonte, USA). The volatile compounds were separated on a DB-5MS column (J & W Scientific Inc., Folsom, USA) (30 m × 0.25 mm *i.d*. × 0.25 μm film thickness) equipped with a Shimadzu QP2010 gas chromatography-mass spectrometry (GC-MS) apparatus (Shimadzu Co., Kyoto, Japan). The initial temperature of the column was 35 °C, which was increased to a final temperature of 240 °C at the rate of 15 °C min^−1^ and held at 240 °C for 4.5 min.

### Protein extraction and LC-MS/MS analysis

Assays were conducted with strawberries immediately at harvest and after 6 d of storage at the simulated shelf-life temperature (20 ± 1 °C) with three independent replications. Proteins were extracted and purified from frozen ground strawberry samples applied with CPPU at both 10 mg L^−1^ and 0 mg L^−1^ (control), using the phenol protocol followed by ammonium acetate–methanol precipitation^17^. Reduced and alkylated protein was then digested by the sequencing-grade modified trypsin (Catalog# V5111; Promega, Madison, WI) at 37 °C. The total peptides were dissolved in a 2% acetonitrile (ACN) and 0.1% trifluoroacetic acid (TFA) for LC-MS/MS analysis. Digested peptides were desalted and separated by reversed-phase chromatography with a nano-HPLC system with a Capillary C18 column (5 μm particle, 150 μm × 10 mm; CTICAP5150100, Column Technology Inc.). A binary solvent gradient was employed: solution A was composed of 0.1% formic acid; solution B was composed of 100% ACN and 0.1% formic acid. The gradient procedure was conducted as follows: 5% solution B for 15 min, increased from 5% to 32% solution B over 45 min, increased to 90% solution B in 35 min, decreased from 90% to 5% solution B in 5 min, and held for 20 min. Separated peptides were analyzed in a LTQ XL mass spectrometer (Thermo Fisher) with a Michrom captive spray nano-electrospray ionization (NSI) source at a flow rate of 2 μl min^−1^. MS and MS/MS spectra were acquired and scans were obtained for the *m/z* range of 400–1800 Da at 50 000 resolution, with the ten most abundant ions in the MS scan selected for automated low energy collision-induced dissociation (CID) whereas the 30 s exclusion time, repeat count of 2, and normalized collision energy of 35% were used for the fragmentation.

### Protein identification and data validation

Raw MS/MS data were searched against NCBI *Viridiplantae* entries, a total of 278 115 sequences that were last updated on Dec. 31, 2011 (NIH, Bethesda, MD, USA) by the MASCOT algorithm version 2.3.02 (Matrix Science, London, UK). The MS and MS/MS mass tolerances were 3.0 and 1.0 Da, respectively, and up to two missed cleavages were permitted for fully tryptic peptides. Carboxamidomethyl cysteine and oxidized methionine were set as fixed and variable modifications, respectively. The false discovery rate (FDR) was determined by using a target-decoy search strategy^50^. PepDistiller^51^, which facilitates the sensitive and accurate validation of MASCOT search results, was used to validate MS/MS-based peptides and the peptide FDR was controlled at 1.0%. A label-free quantification based on the spectral count (SC) was implemented using the SILVER tool developed at the Beijing Proteome Research Center (BPRC)^52^. The relative protein ratio of any proteins found between the groups was calculated by comparing the average abundance values of the protein in each group. Abundance changes above twofold, and *p*-values below 0.05 were used as thresholds to the identified proteins.

### Real-time quantitative PCR (RT-qPCR) analysis

The transcriptomic level of genes in the present study was quantified by reverse transcription followed by RT-qPCR. The target genes used for qPCR were selected on the basis of the protein profiles identified and quantified in Supplementary Table S2. Therefore, FFase, PGM, FBA, Enolase, RubisCO, CS, Acon and MDH involved in the primary metabolism, PDC, ACC, AAT ADH in the pathway of volatile biosynthesis as well as CFI, LDOX, GPX, APX, MDAR and DHAR genes which were related to the stress response system in strawberry were selected for qPCR study. Total RNA was isolated the hot borate protocol reported by Wan and Wilkins^53^. The resulting RNA extracts treated with DNase I and the first-strand cDNA was synthesized. RT-qPCR was performed with an ABI 7500 Real-Time PCR System (Applied Biosystems, USA). An initial hot start was performed at 95 °C for 10 min, followed by 40 cycles of 95 °C for 30 s, 58 °C to 60 °C for 1 min, and 72 °C for 1 min. Expression levels were normalized using the *C*_*T*_ value against the expression level of the actin gene. All gene-specific primers pairs for the qPCR designed using Primer Express 3.0 (Applied Biosystems, Foster City, CA, USA) are listed in Supplementary Table S3. The relative expression levels of the target genes were calculated with formula 2^−ΔΔCt^ 54 and three independent replicates were performed.

### Statistical analysis

All samplings and experiments were conducted as a randomized block design with three technical replicates and three harvests over time as biological replicates. Data were analyzed by ANOVA with the SAS statistical software. The least significant difference (LSD) was calculated to determine significant differences at the 5% level unless stated otherwise. The average from replicates of proteomic and transcriptomic datasets were normalized to log 2 ratios and processed by cluster software (http://biit.cs.ut.ee/clustvis/), which allowed for a complete linkage hierarchical clustering (Euclidean distance) demonstrated with Pearson’s distance and Ward’s algorithm used for data aggregation.

## Additional Information

**How to cite this article**: Li, L. *et al.* Proteomic Response and Quality Maintenance in Postharvest Fruit of Strawberry (*Fragaria* × *ananassa*) to Exogenous Cytokinin. *Sci. Rep.* **6**, 27094; doi: 10.1038/srep27094 (2016).

## Supplementary Material

Supplementary Information

## Figures and Tables

**Table 1:** Periodic residue and dissipation of CPPU in strawberry fruits.

Time after treatment (d)	Average residues (mg kg^−1^)		
	5 mg L^−1^ CPPU	10 mg L^−1^ CPPU	15 mg L^−1^ CPPU
0	0.30 ± 0.04	0.47 ± 0.07	0.64 ± 0.06
3	0.19 ± 0.03^a^ (37.4)^b^	0.33 ± 0.02 (30.5)	0.51 ± 0.04 (19.9)
7	0.14 ± 0.03 (53.8)	0.24 ± 0.02 (48.9)	0.43 ± 0.03 (33.5)
10	0.05 ± 0.00 (84.9)	0.15 ± 0.01 (69.2)	0.30 ± 0.05 (52.5)
15	<MRL^c^	0.05 ± 0.01 (88.9)	0.11 ± 0.02 (83.6)
20	<MRL	0.01 ± 0.00 (97.7)	0.04 ± 0.01 (93.5)
25	<MRL	<MRL	0.02 ± 0.00 (97.2)
30	<MRL	<MRL	<MRL
Regression equation	y = 0.3673e^−0.201x^	y = 0.7062e^−0.194x^	y = 0.8809e^−0.141x^
Coefficient (R^2^)	0.9635	0.9384	0.9438
Half-life DT50 (d)	4.4	5.6	7.2

^a^Data were shown as means ± standard deviation from three replications.

^b^The figures in parenthesis represent per cent dissipation against the deposit values at time 0.

^c^MRL = Maximum Residue Levels (0.01 mg kg^−1^).
