# Supplementary material for: Proteomic Response and Quality Maintenance in Postharvest Fruit of Strawberry (Fragaria × ananassa) to Exogenous Cytokinin
Source: Sci Rep. 2016 Jun 1;6:27094. doi: 10.1038/srep27094 (PMC4890429; doi:10.1038/srep27094)
Supplement: Supplementary Information [file srep27094-s1.pdf]

Proteomic Response and Quality Maintenance in Postharvest Fruit of Strawberry (*Fragaria* × *ananassa*) to Exogenous Cytokinin

Running title: Cytokinin regulatory metabolism of fruit quality

Li Li[1,§] (lili_1066@163.com)

Dongdong Li[1,§] (leeyiaugsep@126.com)

Zisheng Luo[1,*] (luozisheng@zju.edu.cn)

Xinhong Huang[2] (1057466531@qq.com)

Xihong Li[2] (lixihong606@163.com)

[1]Zhejiang Key Laboratory for Agro-Food Processing, College of Biosystems Engineering and Food Science, Zhejiang University, Hangzhou 310058, People's Republic of China. [2]Key Laboratory of Food Nutrition and Safety (Ministry of Education), Tianjin University of Science and Technology, Tianjin 300457, People's Republic of China

[§] These authors contributed equally to this work.

* To whom correspondence should be addressed. Prof Z. Luo (College of Biosystems Engineering and Food Science, Zhejiang University, Yuhangtang road 866, West Lake District, Hangzhou 310058, People's Republic of China. Telephone: +86-571-88982175; Fax: +86-571-88982175.)

Supplementary figure/table legends

Supplementary Figure S1. Relatively quantitative expression levels of genes involved in primary metabolism in strawberry. The relative expression levels of target genes were calculated with the formula: $2^{-\Delta\Delta Ct}$. Data shown are mean ± standard deviation (n = 3). Different lowercase letters represent statistical significance ($P < 0.05$).

Supplementary Figure S2. Relatively quantitative expression levels of genes involved in the volatile biosynthetic metabolism of strawberry. The relative expression levels of target genes were calculated with the formula: $2^{-\Delta\Delta Ct}$. Data shown are mean ± standard deviation (n = 3). Different lowercase letters represent statistical significance ($P < 0.05$).

Supplementary Figure S3. Relatively quantitative expression levels of genes involved in the stress response metabolism of strawberry. The relative expression levels of target genes were calculated with the formula: $2^{-\Delta\Delta Ct}$. Data shown are mean ± standard deviation (n = 3). Different lowercase letters represent statistical significance ($P < 0.05$).

Supplementary Table S1. Aroma volatiles detected in strawberry at harvest and after storage. Data shown are mean ± standard deviation (n = 3).

Supplementary Table S2. Differentially expressed proteins of the strawberry proteome in response to preharvest CPPU application

Supplementary Table S3. Primers used in this study.

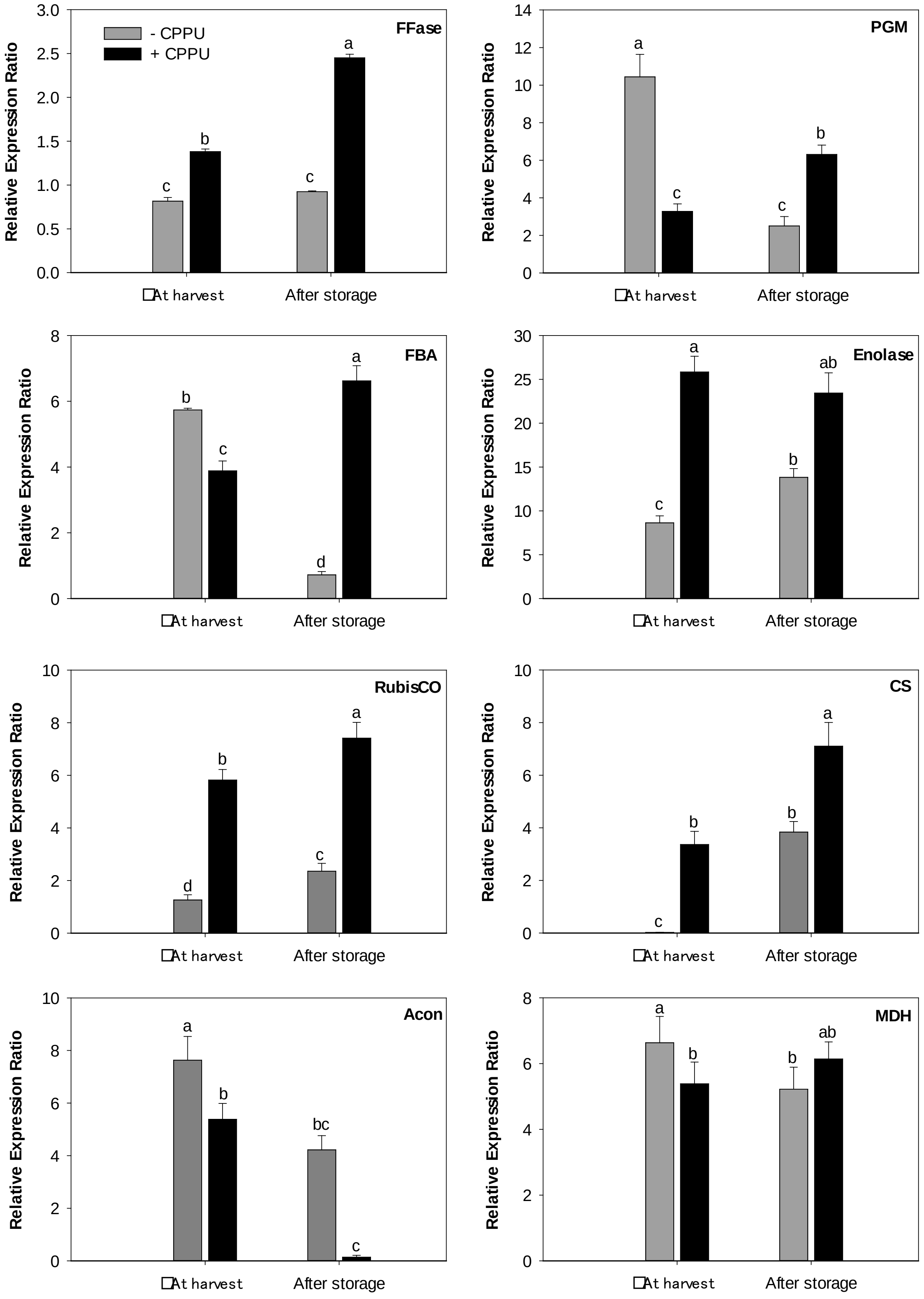

Supplementary Figure S1. Relatively quantitative expression levels of genes involved in primary metabolism in strawberry. The relative expression levels of target genes were calculated with the formula: $2^{-\Delta\Delta Ct}$. Data shown are mean ± standard deviation (n = 3). Different lowercase letters represent statistical significance ($P < 0.05$).

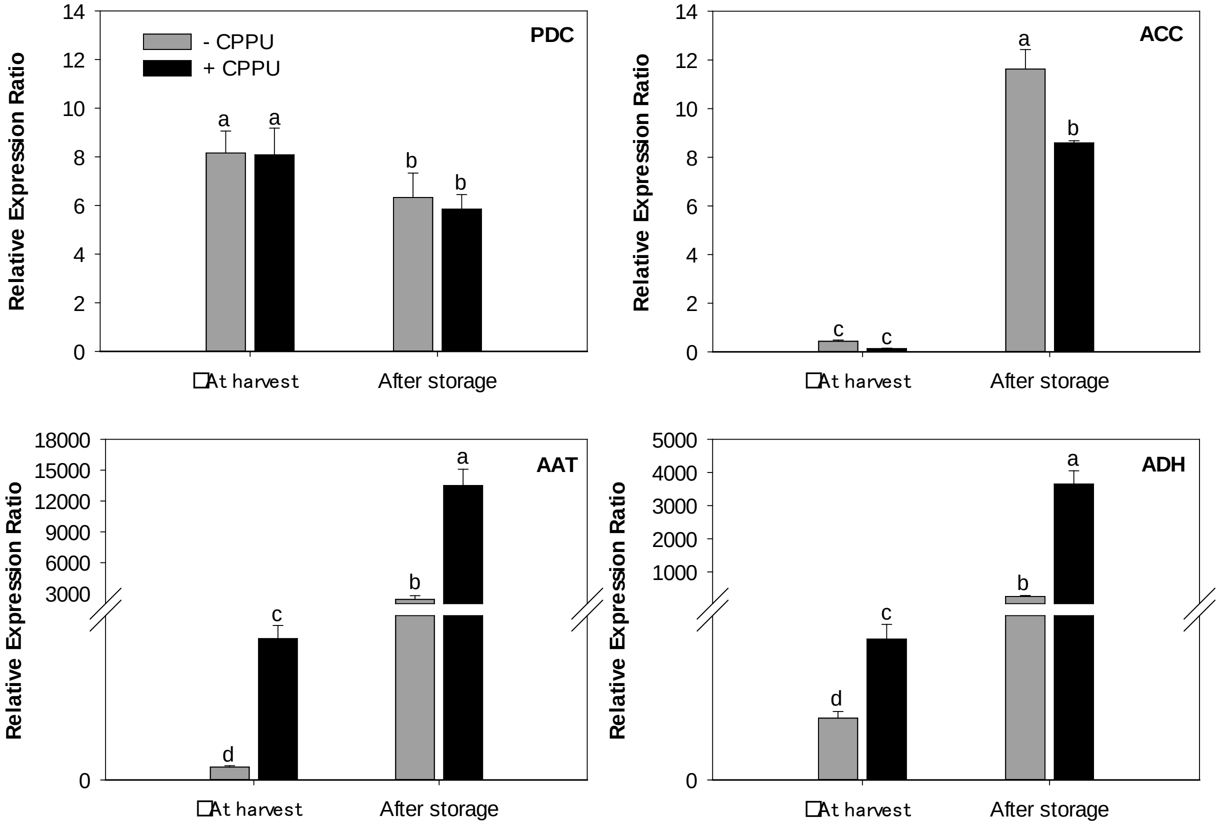

Supplementary Figure S2. Relatively quantitative expression levels of genes involved in the volatile biosynthetic metabolism of strawberry. The relative expression levels of target genes were calculated with the formula: $2^{-\Delta\Delta Ct}$. Data shown are mean ± standard deviation (*n* = 3). Different lowercase letters represent statistical significance ($P < 0.05$).

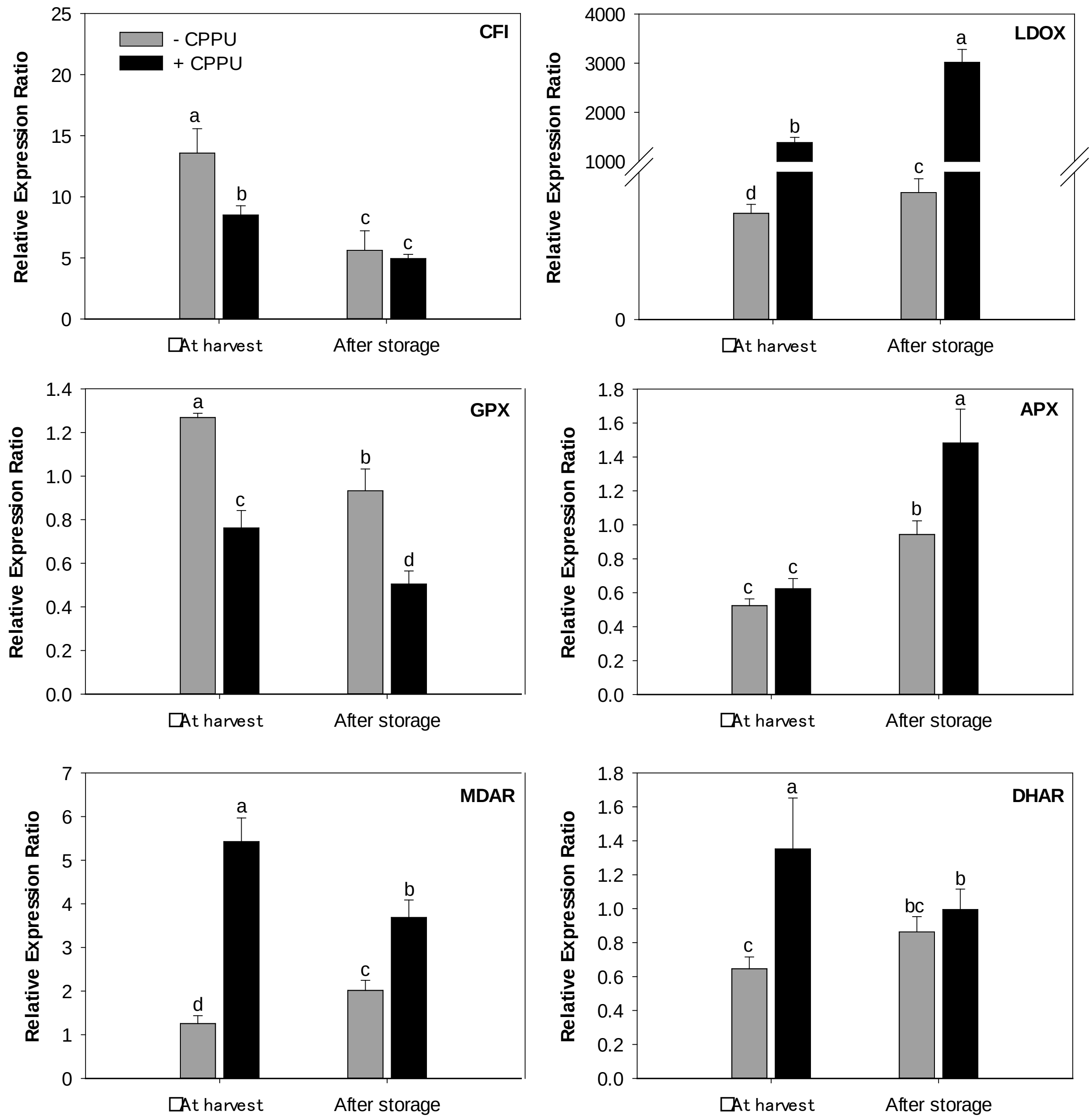

Supplementary Figure S3. Relatively quantitative expression levels of genes involved in the stress response metabolism of strawberry. The relative expression levels of target genes were calculated with the formula: $2^{-\Delta\Delta Ct}$. Data shown are mean ± standard deviation (*n* = 3). Different lowercase letters represent statistical significance ($P < 0.05$).

Supplementary Table S1. Aroma volatiles detected in strawberry at harvest and after storage. Data shown are mean ± standard deviation (*n* = 3).

	Odor description[a]	RI[b]	RI[c]	Retention time (min)	At harvest		After storage	
					–CPPU	+CPPU	–CPPU	+CPPU
**Esters**								
Methyl butyrate	Fruity, sweet	723	729	4.292	0.00[d] ± 0.00	0.43 ± 0.04	0.00 ± 0.00	3.07 ± 0.25
Methyl 3-methylbutanoate	Fruity, Apple-like odor	767		5.647	0.00 ± 0.00	0.00 ± 0.00	0.00 ± 0.00	0.33 ± 0.02
Ethyl hydroxyhexanoate	Fresh	1329		6.351	75.14 ± 3.51	7.43 ± 0.65	25.94 ± 1.36	13.79 ± 2.02
2-butoxyethanol acetate		1090		7.581	10.12 ± 0.95	0.49 ± 0.05	0.36 ± 0.05	0.29 ± 0.01
Ethyl 2-methylbutanoate	Sweet, fruity, strawberry	820		7.962	5.95 ± 0.27	0.50 ± 0.02	0.57 ± 0.04	0.48 ± 0.02
Ethyl valerate	Fruity, orange	871	901	9.87	1.03 ± 0.08	0.52 ± 0.03	1.50 ± 0.02	14.13 ± 0.87
3-methyl pentanoate	Sweet, Apple-like	946	828	10.78	8.21 ± 0.64	3.67 ± 0.01	52.31 ± 7.23	16.39 ± 1.22
Trans-2-methyl-2-butenoate	Fruit, blackcurrant	715	729	11.36	2.65 ± 0.33	0.23 ± 0.00	0.00 ± 0.00	2.32 ± 0.24
Ethyl Hexanoate	Fruity, strawberry	986	998	13.708	413.39 ± 21.5	34.39 ± 0.62	198.38 ± 11.9	26.49 ± 3.25
Hexyl acetate	Fruity, spicy	1000	1009	14.236	0.00 ± 0.00	0.33 ± 0.04	0.00 ± 0.00	0.41 ± 0.04
Methyl phenylacetate	Honey, Jasmine-like	1273		20.087	9.52 ± 0.84	2.10 ± 0.32	25.01 ± 0.36	6.46 ± 0.55
Ethyl Benzoate	Chamomile flower, Fruity	1145	1173	20.386	2.91 ± 0.43	0.20 ± 0.02	1.14 ± 0.20	0.00 ± 0.00
Ethyl octanoate	Fruity, Baked fruity, Sweet	1183	1197	21.231	19.35 ± 2.71	1.40 ± 0.11	14.64 ± 1.82	1.02 ± 0.03
3-hydroxy-4-methyl-ethyl-pentanoate		1412	--	25.937	2.33 ± 0.50	0.09 ± 0.01	0.74 ± 0.11	0.00 ± 0.00
Ethyl cinnamate	Sweet, honey, fruity	1462	1467	30.479	36.07 ± 4.56	0.00 ± 0.00	8.20 ± 0.60	0.00 ± 0.00
Ethyl dodecanoate	Mango-like	1577	1595	33.997	2.53 ± 0.38	0.30 ± 0.02	0.61 ± 0.37	0.00 ± 0.00
**Acids**								
3-Heptenoic Acid		1081		13.954	0.00 ± 0.00	0.30 ± 0.03	0.00 ± 0.00	0.00 ± 0.00
Trans-2-hexenoic acid	Must, fat	1047		15.507	13.59 ± 1.66	0.75 ± 0.06	4.36 ± 0.52	1.84 ± 0.23
Octanoic acid	Sweet, cheese	1158	--	20.502	0.00 ± 0.00	0.00 ± 0.00	0.00 ± 0.00	1.65 ± 0.19
**Alcohols**								

1-methylcyclohexanol		918	952	9.498	0.00 ± 0.00	0.76 ± 0.05	0.00 ± 0.00	0.00 ± 0.00
cis-Linalool oxide	Sweet, woody, floral	1076	1137	17.173	7.35 ± 0.89	1.56 ± 0.96	4.28 ± 0.26	2.16 ± 0.33
Linalool	Muscat, sweet, fruity	1109	1097	17.695	127.97 ± 9.31	34.67 ± 4.25	66.07 ± 0.79	27.07 ± 3.21
Cis-3-nonylene-1-ol		1054	-	17.864	5.15 ± 0.74	0.81 ± 0.07	2.48 ± 0.32	0.78 ± 0.26
2-(4-methyl-3-cyclohexenyl)-2-propanol		1077	-	21.321	24.99 ± 2.12	5.18 ± 0.50	18.56 ± 1.31	6.90 ± 0.31
1,10-decanediol		1518	1549	21.634	2.68 ± 0.51	0.45 ± 0.06	1.87 ± 0.25	0.62 ± 0.24
3,7-dimethyl-2,6-octadien-1-ol	Sweet, fruity, citrus-like	1210	1230	23.231	2.79 ± 0.32	0.70 ± 0.05	2.49 ± 0.30	0.49 ± 0.03
3,7,11-trimethyl-1,6,10-dodecatrien-3-ol	Waxy, floral	1550	1563	33.232	290.29 ± 10.5	88.45 ± 6.52	253.02 ± 17.6	141.34 ± 9.62
**Terpenes**								
Limonene	Citrus-like, Fruity	1048	1029	14.894	2.89 ± 0.40	0.60 ± 0.04	2.28 ± 0.23	1.69 ± 0.11
1-caryophyllene		1759	1419	28.943	5.86 ± 0.27	0.32 ± 0.04	2.43 ± 0.18	1.31 ± 0.14
Cis-β-farnesene	Fruity, Citrus-like, Woody	1445	1457	29.859	4.56 ± 0.70	0.91 ± 0.07	1.35 ± 0.13	1.00 ± 0.07
Cis, trans-α-farnesene		1500	1506	31.555	2.96 ± 0.62	0.44 ± 0.03	1.53 ± 0.14	0.83 ± 0.04
**Furanones**								
Mesifurane	Fruity, caramel, green	1031	--	16.029	468.27 ± 5.52	35.49 ± 2.62	469.75 ± 20.8	127.41 ± 8.62
γ-decalactone	Peach, fat	1422	1467	30.442	0.00 ± 0.00	0.29 ± 0.03	0.00 ± 0.00	0.00 ± 0.00
γ-dodecalactone	Sweet, fruit, flower	1720	1678	35.924	98.75 ± 10.22	6.44 ± 0.52	53.64 ± 6.31	11.64 ± 1.26
**Others**								
Trans-2-hexenal	Apple-like, Fruity, Strawberry	827	855	8.172	39.78 ± 4.62	11.41 ± 0.04	17.34 ± 1.62	15.40 ± 0.83
Benzaldehyde	Almond, burnt sugar	965	960	12.365	11.01 ± 1.20	1.82 ± 0.35	4.01 ± 0.43	2.09 ± 0.24
Azulene		1292	1298	20.986	5.25 ± 0.63	1.88 ± 0.23	0.00 ± 0.00	0.00 ± 0.00
Eugenol	Spicy, honey	1355	1359	26.778	7.25 ± 0.36	0.00 ± 0.00	4.98 ± 0.30	1.16 ± 0.15

[a] Odor descriptions according to the Flavornet (www.flavornet.organd) Pherobase databases (www.pherobase.com).

[b] Calculated retention indices using a series of n-alkanes.

[c] Published retention indices on DB-5 column according to Adams (2001) and the Flavornet database (www.flavornet.org).

[d] The concentrations of volatiles compounds identified were presented as means of μg g^{-1} fresh weight of strawberry ± standard deviation from three replications.

Supplementary Table S2 Differentially expressed proteins of the strawberry proteome in response to preharvest CPPU application

No.[a]	Protein name	Accession number[b]	Mass/pI[c]	Mascot score[d]	Matched peptides number	Sequence coverage (%)	Protein Relative abundance[e]
**Cluster 1**							
1	caffeic acid 3-O-methyltransferase-like	gi|470144514	39.80/5.31	231	5	16	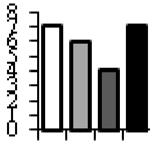
2	vicilin-like antimicrobial peptides 2-2-like	gi|470148689	63.73/5.57	644	5	24	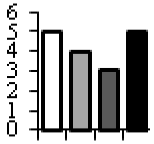
3	annexin D1-like	gi|470103566	36.25/6.43	47	1	8	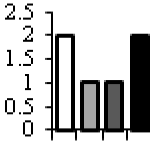
4	uncharacterized protein LOC101294513	gi|470144911	75.22/5.46	245	3	26	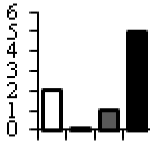
5	uncharacterized protein LOC101292566	gi|470109570	10.81/4.80	661	3	39	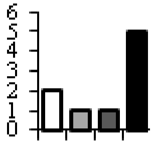
6	calreticulin-like	gi|470132760	47.78/4.43	145	1	8	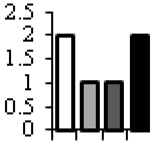
7	proteasome subunit beta type-1-like	gi|470142441	24.38/6.95	88	1	6	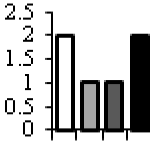
8	translationally-controlled tumor protein homolog	gi|470121591	18.91/4.41	86	2	9	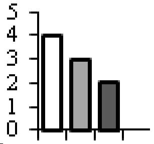

9	V-type proton ATPase subunit E-like	gi|470125749	26.46/8.29	157	2	11	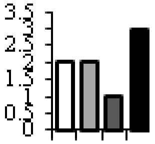
10	glyceraldehyde-3-phosphate dehydrogenase-like	gi|470149016	36.39/8.24	379	7	51	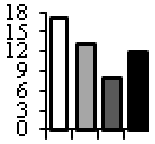
11	ankyrin repeat domain-containing protein 2-like	gi|470107539	38.90/4.44	148	2	11	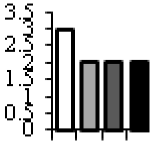
12	metallothionein-like protein 1-like	gi|470118090	6.58/5.07	233	2	15	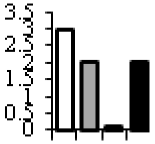
13	5-methyltetrahydropteroyltriglutamate--homocysteine methyltransferase-like	gi|470111481	84.58/6.52	137	2	7	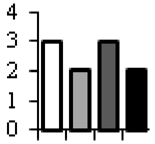
14	alpha-1,4-glucan-protein synthase [UDP-forming]-like	gi|470126624	41.43/6.02	138	2	7	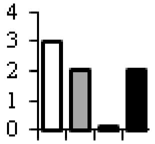
15	triosephosphate isomerase, cytosolic-like	gi|470143704	27.13/6.34	278	4	19	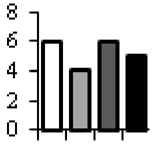
16	probable fructose-bisphosphate aldolase 3, chloroplastic-like	gi|470126093	42.65/8.56	290	3	11	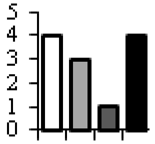
17	pyruvate decarboxylase isozyme 2-like	gi|470133355	65.11/5.91	452	3	22	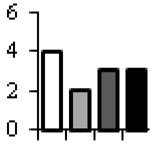
18	probable protein disulfide-isomerase A6-like	gi|470132774	39.67/6.11	65	3	14	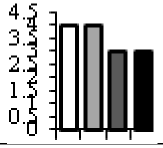

19	major allergen Pru av 1-like	gi|470121687	17.44/5.39	387	5	38	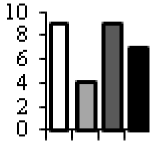
20	60S ribosomal protein L12-like	gi|470110623	17.78/9.00	384	3	22	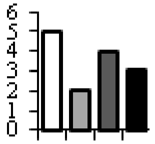
21	heat shock cognate 70 kDa protein-like	gi|470146500	56.91/5.31	163	2	9	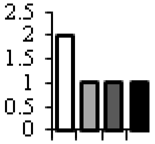
22	glycinin G4-like	gi|470107221	57.89/8.08	354	7	34	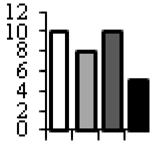
23	luminal-binding protein 5-like	gi|470129154	73.66/5.13	521	4	22	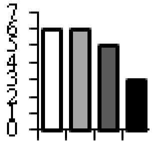
24	elongation factor 1-alpha 1-like	gi|470148685	49.38/9.15	126	4	8	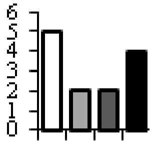
25	peroxiredoxin-2B-like	gi|470128097	17.48/5.34	658	4	20	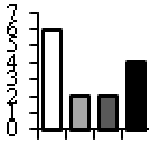
26	26S protease regulatory subunit 10B homolog A-like	gi|470106592	44.78/8.25	119	2	12	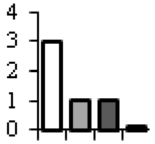
27	probable phospholipid hydroperoxide glutathione peroxidase 6, mitochondrial-like	gi|470125537	25.30/9.31	65	2	6	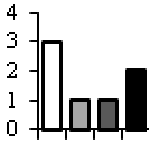
28	40S ribosomal protein S20-2-like	gi|470145076	13.64/9.51	253	2	18	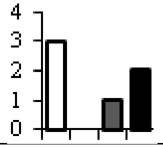

29	14-3-3-like protein-like	gi|470136735	29.71/4.75	568	5	31	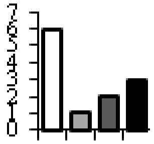
30	eukaryotic translation initiation factor 5A-2-like	gi|470101329	17.39/5.46	231	4	15	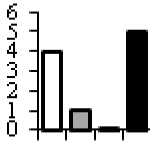
31	ATP synthase subunit beta, mitochondrial-like	gi|470127183	59.13/6.05	134	2	10	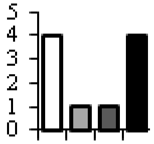
32	chalcone--flavonone isomerase-like	gi|470144168	23.51/4.85	492	3	20	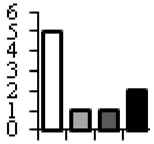
33	phosphoglucomutase, cytoplasmic-like	gi|470122874	62.52/5.74	126	4	14	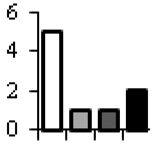
34	ubiquitin-conjugating enzyme E2 10-like isoform 2	gi|470117130	16.48/7.71	358	3	24	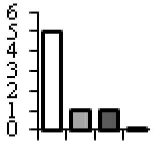
35	cytochrome c oxidase subunit 5b-2, mitochondrial-like isoform 2	gi|470134221	17.71/6.21	258	3	21	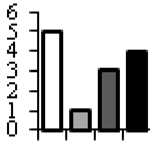
36	glutamine synthetase nodule isozyme-like	gi|470104403	39.05/5.64	88	2	11	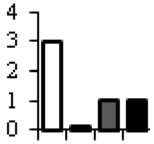
37	probable mediator of RNA polymerase II transcription subunit 37e-like	gi|470133735	70.92/5.11	62	3	5	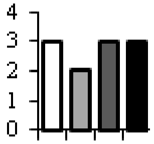
38	uncharacterized protein LOC101306848 isoform 3	gi|470101465	34.80/4.72	185	2	11	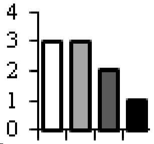

39	uncharacterized protein C167.05-like	gi|470133681	18.14/7.92	46	2	5	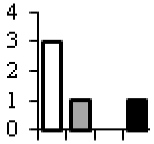
40	pectinesterase/pectinesterase inhibitor U1-like	gi|470135930	62.84/8.77	102	2	13	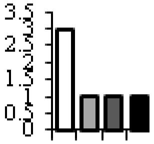
41	aconitate hydratase 2, mitochondrial-like	gi|470103020	108.01/7.08	308	4	8	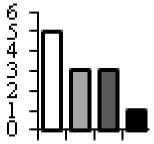
42	cell division cycle protein 48 homolog	gi|470137394	89.52/5.07	57	1	4	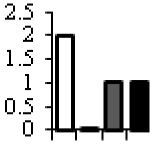
43	flavoprotein WrbA-like	gi|470114574	21.76/5.80	112	1	7	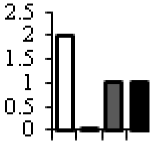
44	adenine phosphoribosyltransferase 1, chloroplastic-like	gi|470109348	27.69/5.83	230	2	9	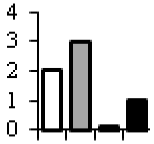
45	actin-depolymerizing factor 3-like isoform 1	gi|470133856	15.81/5.89	142	2	16	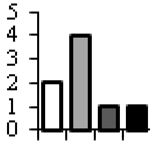
**Cluster 2**							
1	LOW QUALITY PROTEIN: dehydrin COR47-like	gi|470109984	28.56/5.37	85	3	5	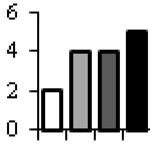
2	biotin carboxyl carrier protein of acetyl-CoA carboxylase 2, chloroplastic-like	gi|470136935	29.54/8.20	45	1	2	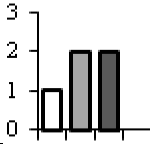

3	leucine aminopeptidase 3, chloroplastic-like	gi|470101341	59.98/7.55	474	5	27	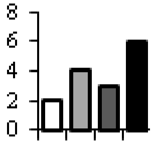
4	26S protease regulatory subunit 6B homolog	gi|470142843	46.03/5.48	163	2	13	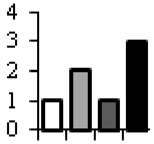
5	cold shock domain-containing protein 4-like	gi|470107029	19.18/5.92	99	2	8	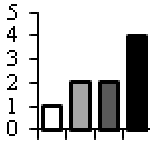
6	uncharacterized protein LOC101314953	gi|470128841	40.70/6.92	372	3	28	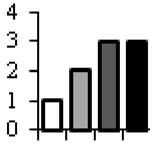
7	dihydrolipoyl dehydrogenase 1, mitochondrial-like	gi|470117795	53.98/6.93	502	5	23	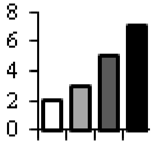
8	tubulin beta-1 chain-like	gi|470116123	50.25/4.74	12	2	9	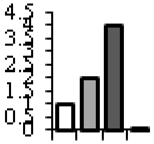
9	staphylococcal nuclease domain-containing protein 1-like	gi|470147845	109.33/7.15	56	1	2	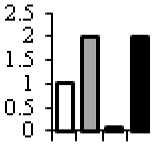
10	uncharacterized protein LOC101298428	gi|470129067	53.14/5.96	83	1	4	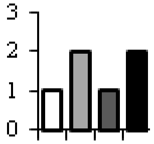
11	12S seed storage protein CRU1-like	gi|470107217	64.49/6.77	366	10	36	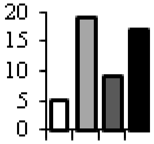
12	thioredoxin H-type-like	gi|470111184	13.35/5.22	179	2	7	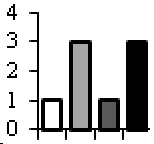

13	60S acidic ribosomal protein P2B-like	gi|470134336	11.45/4.56	57	2	4	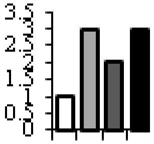
14	ruBisCO large subunit-binding protein subunit beta, chloroplastic-like	gi|470117611	63.75/5.92	54	2	5	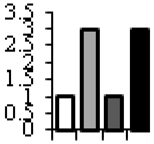
15	protein GrpE-like	gi|470116514	33.22/5.50	273	2	7	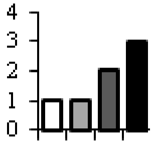
16	transcription factor BTF3-like isoform 1	gi|470126975	18.30/5.79	425	4	37	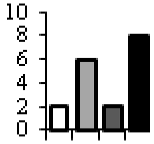
17	probable calcium-binding protein CML13-like	gi|470125402	16.63/5.01	180	2	21	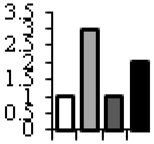
18	heat shock 70 kDa protein, mitochondrial-like	gi|470106955	73.12/5.77	190	3	14	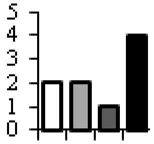
19	legumin B-like	gi|470115872	56.53/6.32	736	20	64	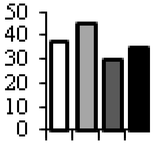
20	legumin A-like	gi|470107215	56.61/6.84	437	15	42	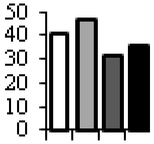
21	peptidyl-prolyl cis-trans isomerase 1-like	gi|470107009	18.27/8.53	**58**	2	3	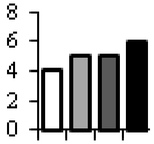
22	uncharacterized protein LOC101304165	gi|470113601	57.00/5.96	332	4	16	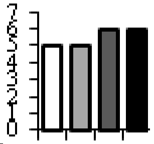

23	malate dehydrogenase, mitochondrial-like	gi|470115832	35.62/8.46	631	8	46	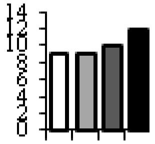
24	enolase-like	gi|470134388	47.94/5.76	426	5	27	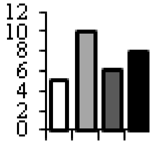
25	>gi|470137735|ref|XP_004304618.1| PREDICTED: quinone oxidoreductase-like protein At1g23740, chloroplastic-like	gi|470137735	34.19/5.72	724	18	72	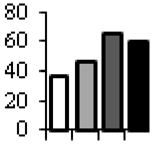
26	cysteine proteinase inhibitor 12-like isoform 1	gi|470143070	26.29/6.17	104	2	9	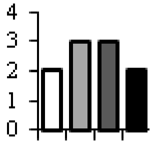
27	12S seed storage protein CRU2-like	gi|470127983	35.82/5.25	358	3	22	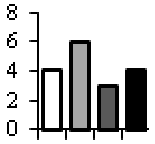
28	leucoanthocyanidin dioxygenase-like	gi|470125461	42.86/5.48	90	2	6	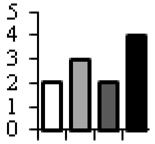
29	non-functional NADPH-dependent codeinone reductase 2-like	gi|470126493	36.14/5.93	389	5	26	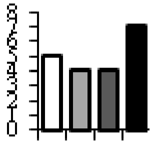
30	L-ascorbate peroxidase, cytosolic-like	gi|470117066	27.28/5.68	520	6	35	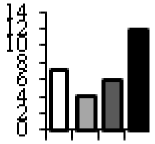
31	uncharacterized protein LOC101299909 isoform 1	gi|470106646	17.17/6.33	235	4	23	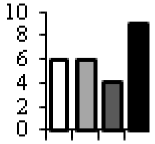
32	malate dehydrogenase, cytoplasmic-like	gi|470120564	35.60/6.01	44	4	41	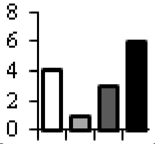

33	phosphoglycerate kinase, cytosolic-like	gi|470141725	42.29/6.20	611	9	43	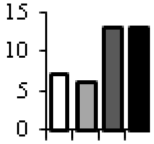
34	vicilin-like antimicrobial peptides 2-1-like	gi|470112718	58.50/6.93	560	7	26	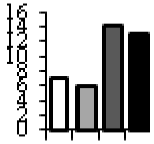
35	protein disulfide-isomerase-like	gi|470109183	55.86/4.94	80	7	35	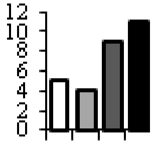
36	nascent polypeptide-associated complex subunit alpha-like protein-like	gi|470141512	21.93/4.30	53	3	7	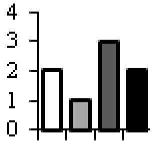
37	26S proteasome non-ATPase regulatory subunit 4-like	gi|470107043	42.74/4.40	317	2	13	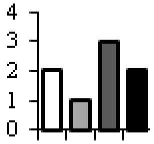
38	legumin A-like	gi|470107219	62.76/8.18	260	6	24	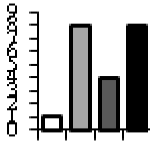
**Cluster 3**							
1	fructose-bisphosphate aldolase cytoplasmic isozyme-like	gi|470134363	38.58/7.61	47	1	3	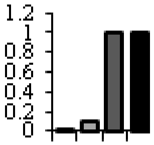
2	mitochondrial-processing peptidase subunit alpha-like	gi|470133998	54.17/5.90	89	1	6	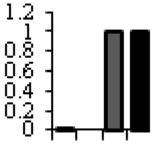
3	monodehydroascorbate reductase-like	gi|470134340	47.12/6.22	71	1	3	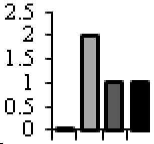

4	uncharacterized protein LOC101293933	gi|470144907	11.39/9.09	148	1	14	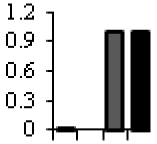
5	20 kDa chaperonin, chloroplastic-like	gi|470102438	26.14/9.03	63	1	9	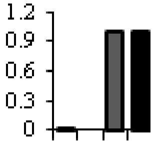
6	dihydrolipoyllysine-residue succinyltransferase component of 2-oxoglutarate dehydrogenase complex 2, mitochondrial-like	gi|470103044	50.79/9.16	101	1	7	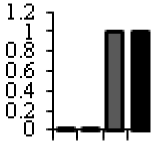
7	histone H2A-like	gi|470116850	16.26/10.66	265	1	11	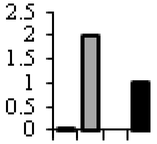
8	non-specific lipid-transfer protein-like isoform 1	gi|470133509	11.66/9.44	42	1	6	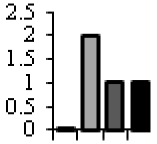
9	unknown protein DS12 from 2D-PAGE of leaf, chloroplastic-like	gi|470142759	31.08/5.19	85	1	5	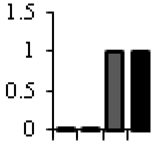
10	peptidyl-prolyl cis-trans isomerase FKBP12-like	gi|470108216	11.96/5.79	93	1	3	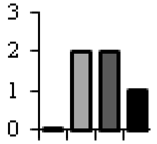
11	peptide methionine sulfoxide reductase-like	gi|470126390	21.54/7.00	326	2	11	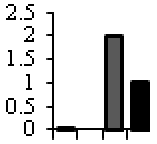
12	obg-like ATPase 1-like	gi|470115595	44.46/6.40	92	1	4	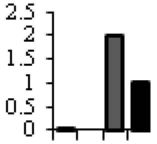
13	uncharacterized protein LOC101290918	gi|470147857	83.55/5.55	55	1	5	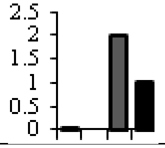

14	40S ribosomal protein S12-like	gi|470101313	15.52/5.29	69	1	7	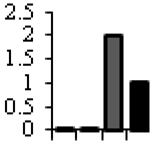
15	dnaJ protein homolog	gi|470110519	46.51/6.19	260	2	12	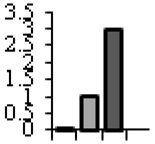
16	dihydrofolate reductase-like	gi|470101485	30.66/6.66	115	1	3	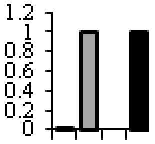
17	polyadenylate-binding protein 2-like	gi|470136476	70.96/8.38	131	1	2	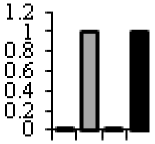
18	proteasome subunit beta type-4-like	gi|470148670	27.22/7.71	268	1	14	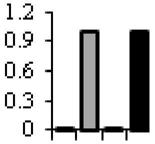
19	inositol-tetrakisphosphate 1-kinase 1-like	gi|470146922	35.45/5.68	82	1	3	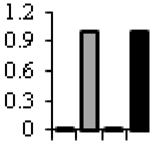
20	UBA and UBX domain-containing protein At4g15410-like	gi|470114245	32.55/5.25	99	1	4	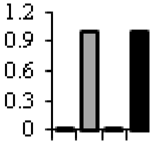
21	V-type proton ATPase subunit G-like isoform 2	gi|470137340	12.17/9.29	75	1	5	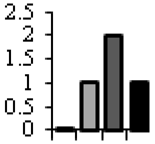
22	heat shock cognate protein 80-like	gi|470117509	80.11/4.97	116	2	11	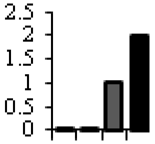
23	heat shock 70 kDa protein 15-like	gi|470134139	93.48/5.11	137	1	5	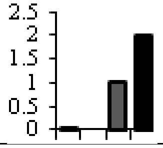

24	thiol protease aleurain-like	gi|470102394	39.42/6.26	85	1	4	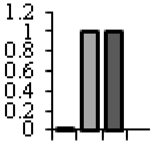
25	endoglucanase 8-like	gi|470122866	54.92/9.11	43	1	3	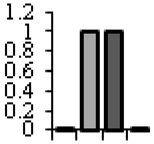
26	dihydrolipoyllysine-residue acetyltransferase component 3 of pyruvate dehydrogenase complex, mitochondrial-like	gi|470135736	58.48/7.60	146	2	8	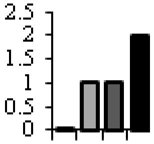
27	mitochondrial outer membrane protein porin 4-like	gi|470120659	29.48/8.95	165	1	5	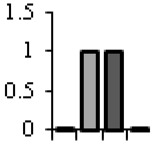
28	21 kDa protein-like	gi|470122868	21.78/6.27	291	1	6	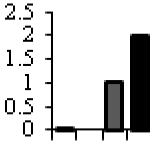
29	aspartic proteinase nepenthesin-2-like	gi|470135396	49.96/8.32	292	1	19	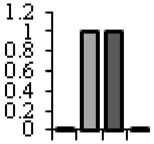
30	beta-fructofuranosidase, insoluble isoenzyme CWINV1-like	gi|470123049	64.79/9.17	350	1	10	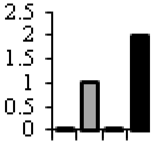
31	late embryogenesis abundant protein 2-like	gi|470134229	17.69/9.36	107	1	9	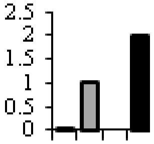
32	citrate synthase, mitochondrial-like isoform 1	gi|470141536	52.06/7.69	68	1	4	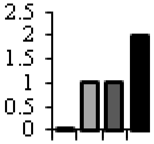
33	uncharacterized protein At2g27730, mitochondrial-like	gi|470113710	10.76/7.91	440	2	36	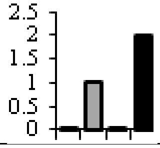

34	C2 domain-containing protein At1g63220-like isoform 1	gi|470142243	28.13/4.87	224	2	12	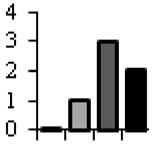
35	adenosine kinase 2-like	gi|470137378	37.33/5.14	63	2	8	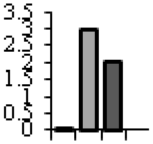
36	uncharacterized protein LOC101302504	gi|525345151	14.89/5.19	107	1	4	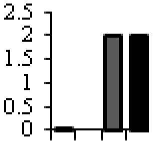
37	proteasome subunit alpha type-2-A-like	gi|470115143	25.52/5.22	81	1	7	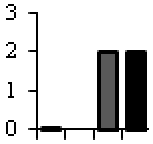
38	40S ribosomal protein S18-like	gi|470126286	17.65/10.56	189	1	5	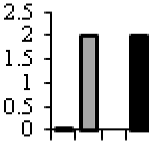
39	acetyl-coenzyme A carboxylase carboxyl transferase subunit alpha, chloroplastic-like	gi|470133575	83.84/8.77	95	2	3	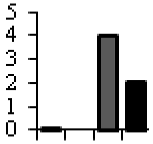
40	1-Cys peroxiredoxin-like	gi|470122127	24.30/6.31	251	3	15	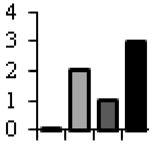
41	malate dehydrogenase, chloroplastic-like	gi|470126765	42.91/6.55	56	2	17	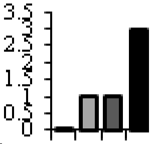

[a] Protein no. and order correspond to the hierarchical clustering analysis in Fig. 4.

[b] Accession numbers from the NCBI *Viridiplantae* database.

[c] Experimental mass (kDa) and isoelectric point (pI) of identified proteins.

[d] Mascot score reported after searching against the NCBI *Viridiplantae* database.

[e] Relative abundances of normalized protein expression; values are expressed as the mean of three replications. White, light grey, dark grey, and black

bars show the abundance in strawberry at harvest without CPPU (AH–CPPU), at harvest with CPPU (AH+CPPU), after storage without CPPU (AS–CPPU), and after storage with CPPU application (AS+CPPU), respectively.

Supplementary Table S3. Primers used in this study.

Gene	Forward (5'–3')	Reverse (5'–3')
FFase	AGGCAAGGAGTGCATAACAG	ATAAGTAGATGGGTCACATACAAGA
PGM	CTCACTTCCTCACCGTCAAA	CCGCTGGTTCCCGTCTT
FBA	GATGTCTTGAACGAGGGTGG	CCTGGCAGATGATAGCATAACG
Enolase	GCTTTGATCGGCAAGGACC	GCAGGAACAGGCAACACCA
RubisCO	GCAGGCTGAAACAGGTGAA	ACGGTGGATGTGAAGAAGTAGA
CS	CTTTGAAGCACTTGCCTGAC	CCCGATACTCCTTGAGACCC
Acon	TTCAACACTAACGGCTTACTCTACC	CCAGCTACTCCCAAACCATC
MDH	GAGAAACCCGTCCGTGAGC	CAACAAGTGACAGGGAATGAGTAA
PDC	CCAAGGTGCGTTGCGAAGA	GGCAGCAGAAACCCTAGACCC
ACC	GATTCCCGCTCCTGTTAGT	CTTGAAGAAGACTCTGCCCTA
AAT	GATGGAGGAACGGCAGAC	TGGCTTGGGCACGGACT
ADH	TGAAGTTACGGAAGTAGGGAGC	AAGAGGCAAGTTGTCTGGGAT
CFI	ACGACAACCTCCACCAAACC	CACGGCCTTATCCTCCAAGTA
LDOX	GCCTCAAACACCTTCCGACTA	TCCAGCCTCCCTTCTTCTAATC
GPX	AAATCCTCACTTGGGTCCTC	GTAGCAGCTCTTGCATAAACAC
APX	AAATACGCTGCGGATGAAGA	AGAAACAACACCAACTGCCTAAA
MDAR	GCTGTGATTGTTGGAGGAGG	GCACCAGGGTTCAGGGTA
DHAR	CCTGAGCCGCCTTTGACA	GGCCCATTTAATTTGAGGTAATC
Actin	TGGGTTTGCTGGAGATGAT	CAGTAGGAGAACTGGGTGC
